# Identification of hybridization and introgression between *Cinnamomum kanehirae* Hayata and *C. camphora* (L.) Presl using genotyping-by-sequencing

**DOI:** 10.1038/s41598-020-72775-0

**Published:** 2020-09-29

**Authors:** Chia-Chen Wu, Shu-Hwa Chang, Chih-Wei Tung, Cheng-Kuen Ho, Yolanda Gogorcena, Fang-Hua Chu

**Affiliations:** 1 Silviculture Division, Taiwan Forestry Research Institute, Council of Agriculture, Executive Yuan, Taipei, Taiwan; 2 School of Forestry and Resource Conservation, National Taiwan University, Taipei, Taiwan; 3 Department of Agronomy, National Taiwan University, Taipei, Taiwan; 4 Laboratory of Genomics, Genetics and Breeding of Fruit Trees and Grapevines, Experimental Station of Aula Dei-CSIC, Zaragoza, Spain

**Keywords:** Biotechnology, Genetics

## Abstract

*Cinnamomum kanehirae* Hayata and *C. camphora* (L.) Presl are important tree species in eastern Asia. The wood of *C. kanehirae* is in increasing demand for culturing *Antrodia cinnamomea*, a medicinal fungus that naturally grows inside the trunk of *C. kanehirae*. Putative hybrids between *C. kanehirae* and *C. camphora* were previously reported but with no scientific evidence, leading to confusion or misplanting. First, to identify the female parent of putative hybrids, the maternal inheritance InDel (insertion/deletion) markers were developed by using low-coverage sequencing. SNPs were developed by using genotyping-by-sequencing (GBS) approach in *C. kanehirae*, *C. camphora* and putative hybrids. The results indicated that the female parent of the studied hybrids was *C. camphora*. Eight hundred and forty of the 529,006 high-density SNPs were selected and used for analysis. Hybrids were classified as F1 (*C. kanehirae* × *C. camphora*), F2 and backcrosses. Hybridization has occurred in the human-developed area of eastern and southwestern Taiwan, and the introgression was bidirectional. For producing pure wood, buffering zones should be established around seed orchards to avoid cross-species pollination and to preserve the genetic purity of *C. kanehirae*. The DNA markers developed in this study will also be valuable for further wood identification, breeding and evolutionary research.

## Introduction

Hybridization occurs when mating between individuals from interspecific species generates viable offspring, and this process is estimated to occur in approximately 25% of all plant species^1^. Introgression may also occur when F1 hybrids backcross to one or both parental species, leading to foreign genetic material being integrated into the parent genome^2^. Diversification and speciation may also be caused by hybridization and introgression if offspring undergo reproductive isolation or have increased fitness. Moreover, hybridization may affect growth vigor, and as a consequence, the wood quality may change, with negative effects on parasitic species growing in the wood^3^. In the past, the morphological characteristics have been the only method available for classifying hybrids; however, morphological characteristics alone may not be sufficient to identify hybrids. Therefore, molecular markers provide a good tool for estimating the gene flow and genetic structure of hybrids^4^. Since the inheritance modes of the chloroplast/mitochondria and nuclear DNA are distinct, a combination of nuclear and chloroplast/mitochondria markers can be used to understand the complex mode of gene flow in hybrids. For instance, maternally inherited chloroplast/mitochondrial DNA markers can be used to assess the maternal parent of the hybrids and the direction of hybrid mating^2^.

Genotyping-by-sequencing (GBS) is a direct sequencing approach based on the high-throughput next-generation sequencing (NGS) of genomic subsets targeted by restriction enzymes^5^. This approach can be generalized for any plant species at a low per-sample cost, providing an incredible number of markers. GBS can be applied for genotyping the variance of individuals and is also suitable for population studies, germplasm characterization and assisted breeding in diverse organisms. InDel and SNP (single nucleotide polymorphism) markers have received increasing attention due to their many advantages, such as high polymorphism, codominance, high stability and high density. Recently, InDel and SNP markers have been used for genetic and evolutionary studies in many plant species^6–8^.

Taiwan is located in the middle of the western Pacific Ocean and has very high biodiversity. Due to its geographical isolation, hundreds of plant species are endemic to Taiwan. *Cinnamomum* is one genus in the Lauraceae family, and it is composed of approximately 250 species growing in tropical and subtropical regions in eastern Asia, Australia and the Pacific islands^9,10^. According to Huang^11^, there are twelve native *Cinnamomum* species in Taiwan. *C. kanehirae* is an endemic and very valuable tree species in Taiwan. Its wood is in high demand due to its unique usage for culturing the naturally parasitized fungus *Antrodia cinnamomea* Chang and Chou. This fungus specifically parasitizes inside of the *C. kanehirae* wood and has been used as a Chinese medicine known to have many pharmacological effects, with prices above 10,000 US$ per kilogram^12–14^. However, *C. kanehirae* wood could be wrongly identified by using only morphological methods. We previously successfully identified the wood of *C. kanehirae* and *C. micranthum* by using chloroplast and chemical markers^6^. Recently, it was reported that some *C. kanehirae* seedlings may actually be hybrids with other *Cinnamomum* species^15,16^. It has been reported that putative hybrids are found, though rarely, in non-natural habitats such as nurseries, seed orchards and gardens. These hybrids can cause confusion for growers, and thus, a reliable method of hybrid identification is needed. The metabolite profiles and pharmacological effects of *A*. *cinnamomea* cultivated in the *C. kanehirae* wood are completely different by using different wood species substrates^6,13^. In addition, according to Taiwanese regulations, all *A. cinnamomea* products must be obviously labeled with the culturing methods and produce processing on the package. Even in China, all of the *A. cinnamomea* medicine must be cultured on *C. kanehirae* wood. Therefore, it is very important to identify the correct *C. kanehirae* species and clearly differentiate it from its hybrids. However, we speculate that some morphological traits of hybrids are different from those of either *C. kanehirae* or *C. camphora*. Even the metabolite profile of *A. cinnamomea* cultured by using hybrid wood may be different from that using genuine *C. kanehirae* wood. In the aspect of management, the putative hybrids will lead to worrisome issues that may spoil pure *C. kanehirae* and affect the genetic pool of the natural populations of *C. camphora* and *C. kanehirae*. Moreover, hybridization may lead to genetic swamping, where hybrids overwhelm or outcompete the rarer parent species^17^. To avoid the loss of *C. kanehirae* genetic diversity and the misuse of hybrid woods, it is very important to further understand hybrids between these two species in the wild.

Genomic research of *C. kanehirae* has improved with the recent release of the complete chloroplast genome and whole-genome sequencing data^6,18,19^. In the present paper, we applied GBS and a genome reference-based method for calling SNP data in 75 individuals, namely, 19 *C. kanehirae*, 31 *C. camphora* and 25 putative hybrids, in order to prove the occurrence of hybridization as well as the frequency and direction of hybridization. We also developed and validated the InDel markers by using low-coverage sequencing of interspecies and the GATK (Genome Analysis Toolkit) pipeline to determine which species is the putative donor parent in cases of hybridization.

This is the first report of SNP genotyping in *C. kanehirae* and *C. camphora*. The genetic analysis of these two *Cinnamomum* species and hybrids could provide evidence for taxonomy, practical molecular biomarkers for wood identification and recommendations for wild resource management in the future.

## Results

### The validation of InDel markers

**Figure 1:**
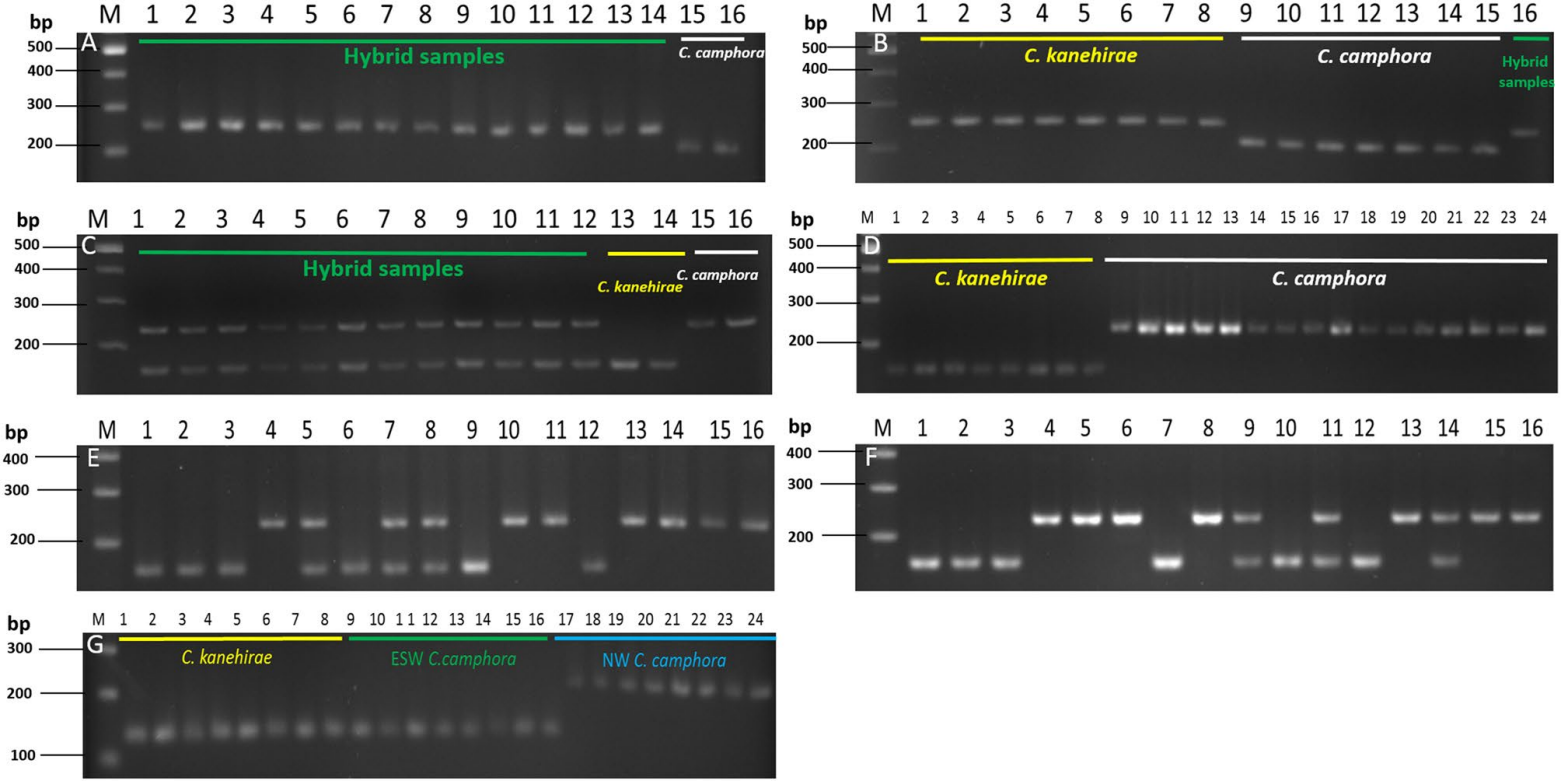
The gel electrophoresis of InDel, ZS_CK InDel_1 (mitochondrial DNA) (**A,B**), JLH2_CK InDel_4 (codominant) (**C**–**F**) and ZS_CK InDel_2 (chloroplast DNA) (**G**). M: 100 bp DNA ladder. (**A**) Lanes 1–14: hybrids, Lanes 15–16: *C. camphora*. (**B**) Lanes 1–8: *C. kanehirae*, Lanes 9–15: *C. camphora*, Lane 16: hybrid. (**C**) Lanes 1–12: hybrids, Lanes 13 and 14: *C. kanehirae*, Lanes 15 and 16: *C. camphora*. (**D**) Lanes 1–8: *C. kanehirae*, Lanes 10–24: *C. camphora*. (**E**) Lane 1: *C. kanehirae*, Lanes 2–14: hybrids, Lanes 15 and 16: *C. camphora*. (**F**) Lane 1: *C. kanehirae*, Lanes 2–14: hybrids, Lane 15 and 16: *C. camphora*. (**G**) Lanes 1–8: *C. kanehirae*, Lanes 9–16: ESW *C. camphora*. Lanes 17–24: NW *C. camphora*. The used samples are listed in Supplementary Table S5 online.

Low-coverage sequencing was performed in three individuals of two *Cinnamomum* species: *C. kanehirae* (S99), *C. camphora* (JLH2) and *C. camphora* (JZS). Statistical data from this low-coverage sequencing are available in Supplementary Table S1 online. In *C. kanehirae*, we obtained the greatest number of clean bases after trimming. In total, we found 481 InDels that satisfied our filtering parameters. We validated the five longest InDels in three data sets. Finally, 7 out of 15 validated InDels (46.67%) were polymorphic in *C. kanehirae* and *C. camphora*. Among the 7 InDels, 1 had blastn matches to nuclear mRNA, 2 had blastn matches to mitochondrial sequence, 1 had blastn matches to chloroplast sequence, and 3 had no blastn matches (see Supplementary Table S2 online). The InDel marker named ZS_CK InDel_1 is annotated to mitochondrial DNA. All of the genotypes of ZS_CK InDel_1 in putative hybrid samples showed the same profile as *C. kanehirae*, different from the *C. camphora* profile (Fig. 1A,B). However, the marker JLH2_CK InDel_4 is recognized as a nuclear and codominant marker, and its profile in all putative hybrids (F1) showed two clear double bands (152 bp and 227 bp), where one DNA fragment is identical to *C. kanehirae* (152 bp) and the other DNA fragment is identical to *C. camphora* (227 bp) (Fig. 1C,D). However, three genotypes were obtained (152 bp, 227 bp, or 152 and 227 bp) in other putative hybrids (F2, B × 0 and B × 1, see Fig. 1E,F). Since the individuals were classified as F2 or backcrosses, which underwent genomic recombination when crossed, the codominant nuclear genes will show a biparental or uniparental genotype. In the present InDel data, we also found that the populations of *C. camphora* showed different genotypes in the chloroplast InDel (ZS_CK InDel_2) (Fig. 1G). The *C. camphora* can be clustered into two geographical collection areas: the ESW (east and southwest) and NW (north and west, including Japan and Vietnam) populations. The genotyping of *C. kanehirae* and ESW *C. camphora* are identical (124 bp) but different from that of NW *C. camphora* (212 bp). This is in accordance with other studies, which observed both kinds of chloroplast sequences in *C. camphora* (NCBI accession numbers: MF421523 and MG021326).

### Genetic diversity and SNP variation

The number of sequencing reads was 267,249,305 with 40,354,645,055 base pairs in GBS (see Supplementary Fig. S1 online). In total, 529,006 SNPs were obtained in the calling analysis. However, the proportions of missing sites in *C. kanehirae*, *C. camphora* and putative hybrids were 0.36–0.72, 0.39–0.95 and 0.33–0.54, respectively. Since the site of missing data could be relative to InDel or low-quality sequencing, we trimmed off the sites with missing data in all of the samples. Finally, 840 SNPs were obtained for further analysis (see Supplementary Fig. S1 online). All 840 loci were polymorphic in 75 samples: 278 SNPs in *C. kanehirae*, 201 in ESW *C. camphora*, 376 in NW *C. camphora* and 431 in putative hybrids are polymorphic (Table 1). Twenty-one SNPs in *C. kanehirae*, 0 in ESW *C. camphora*, 12 in NW *C. camphora* and 4 in putative hybrids significantly deviated from Hardy–Weinberg equilibrium (P < 0.0001)^20^. This may be due to genotyping errors, inbreeding, selection and population stratification. In genomic studies, the SNPs that are not consistent with Hardy–Weinberg equilibrium need to be removed from further analysis^21^. Thus, 32 SNPs were removed, and 808 SNPs were used for further analysis. The expected heterozygosity (*He*) was estimated in each group: 0.084 ± 0.005 (*C. kanehirae*), 0.076 ± 0.005 (ESW *C. camphora*), 0.123 ± 0.006 (NW *C. camphora*) and 0.143 ± 0.007 (putative hybrids). The *F*_*is*_ (inbreeding coefficient) values were 0.102, − 0.119, 0.118 and − 0.099 in *C. kanehirae*, ESW *C. camphora*, NW *C. camphora* and putative hybrids, respectively (Table 1). *F*_*st*_ was estimated to be 0.642 between *C. kanehirae* and ESW *C. camphora*, 0.560 between *C. kanehirae* and NW *C. camphora*, and 0.304 between *C. kanehirae* and hybrids. The *F*_*st*_ between ESW and NW *C. camphora* was estimated to be 0.240 (Table 2). P values were based on 9,999 permutations and are significant (P < 0.0001).

**Table 1:** Genetic diversity of all samples: *C. kanehirae*, *C. camphora* collected in ESW and NW and putative hybrids.

	75 samples	*C. kanehirae*	ESW *C. camphora*	NW *C. camphora*	Putative hybrid
Polymorphic SNP loci	840	278	201	376	431
SNP loci significantly deviated from Hardy–Weinberg equilibrium (P < 0.0001)	32	21	0	12	4
Ho	–	0.076 ± 0.005	0.085 ± 0.006	0.108 ± 0.006	0.158 ± 0.008
He	–	0.084 ± 0.005	0.076 ± 0.005	0.123 ± 0.006	0.143 ± 0.007
*F*_*is*_	–	0.102	− 0.119	0.118	− 0.099

Observed Heterozygosity (Ho) = No. of Hets/N, Expected Heterozygosity (He) = 1 − Sum pi^2^, Fis = (He − Ho)/He.

**Table 2:** Genetic differentiation of *C. kanehirae*, ESW and NW *C. camphora* and putative hybrids (Lower diagonal: pairwise *F*_*st*_; P values based on 9999 permutations are shown above the diagonal).

	*C. kanehirae*	ESW *C. camphora*	NW *C. camphora*	Hybrid
*C. kanehirae*	–	0.0001	0.0001	0.0001
ESW *C. camphora*	0.642	–	0.0001	0.0001
NW *C. camphora*	0.560	0.240	–	0.0001
Hybrid	0.304	0.208	0.267	–

### Phenotypic analysis, genetic structure and hybrid classification

In the morphological trait analysis, we collected and measured 229, 109 and 109 fruits from *C. camphora*, *C. kanehirae* and putative hybrids, respectively. The back-leaf properties of these samples were also recorded and described. All of the putative hybrids have oblate shaped fruits, as do *C. kanehirae*, and the back-leaf is covered with a powder-like white wax but less noticeably than in *C. camphora* leaves (see Supplementary Fig. S2 online). The ratios of the fruit length and width (L/W ratio) are available in Supplementary Table S3 online. The results indicated that the L/W ratios are 0.762, 0.867 and 0.992 in *C. kanehirae*, putative hybrids and *C. camphora*, respectively. The fruit shape of putative hybrids and *C. kanehirae* is oblate, while the fruits of *C. camphora* are almost round. The fruits of *C. kanehirae* and putative hybrids are similar in shape and larger than those of *C. camphora*. The differences in the fruit shapes of *C. kanehirae* and *C. camphora* have been qualitatively described in previous publications^22,23^. The characteristics of the back-leaf in putative hybrids are intermediate between those of *C. kanehirae* (glossy and bright) and *C. camphora* (covered with a powder-like white wax). The individual putative hybrids are seedlings of fruits from the *C. kanehirae*. Thus, we could infer based on morphological traits that the putative mother trees are *C. kanehirae* and the putative male ancestor may be related to *C. camphora.* However, no molecular evidence was used previously to illustrate the relationship of hybridization and the level of introgression of *C. camphora* and *C. kanehirae*.

**Figure 2:**
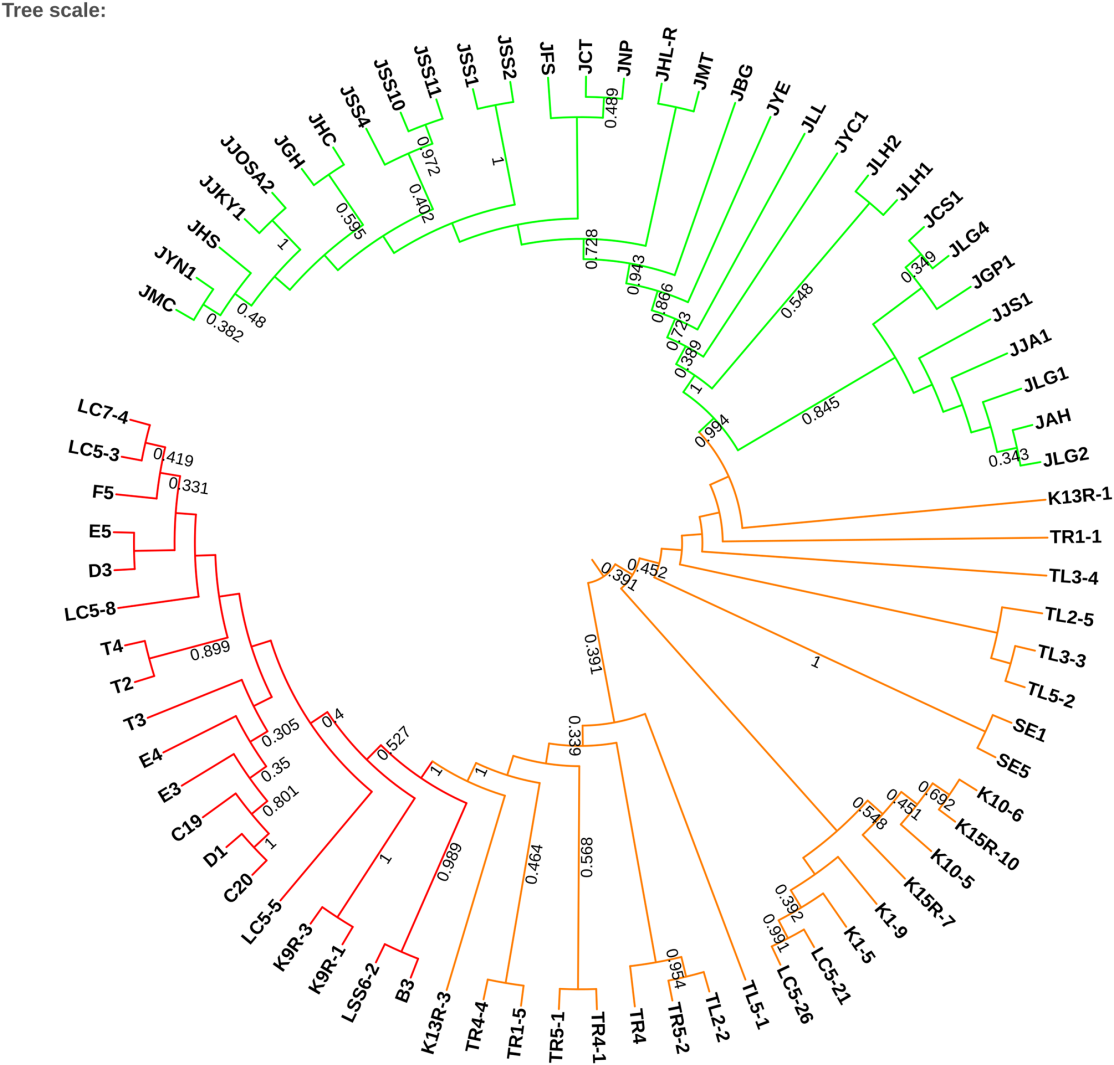
Neighbor-joining tree based on 808 SNPs. Bootstrap values from 1000 replicates are shown on the nodes. Colors on branches are colored by clusters. *C. kanehirae* is in red, putative hybrids are in orange, and *C. camphora* is in green.

**Figure 3:**
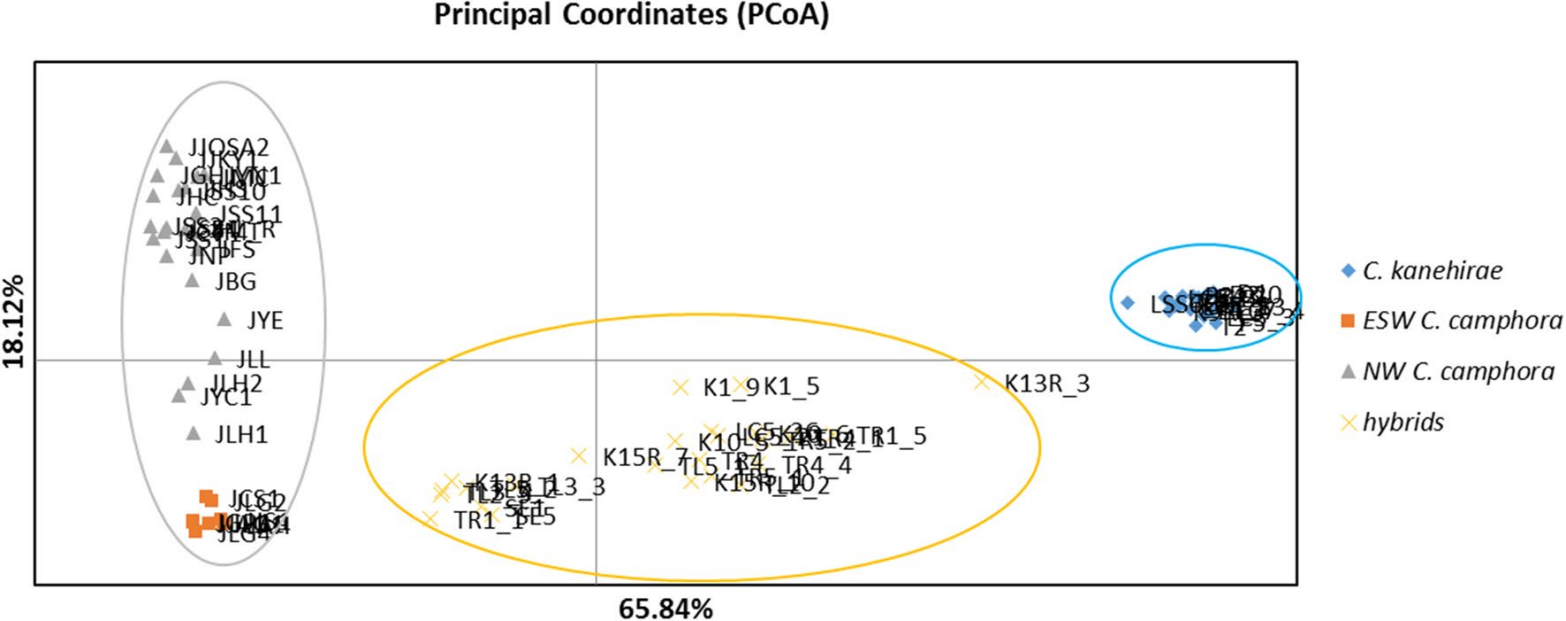
Principal coordinates analysis (PCoA) based on the Nei’s pairwise genetic distances of *C. kanehirae*, *C. camphora* and hybrids (75 samples). Colored symbols inside each oval are represented as in the legend. Samples are as in Supplementary Table S4 online.

The neighbor-joining tree of 808 SPNs was constructed with 1000 bootstraps (Fig. 2). It clearly shows that *C. kanehirae*, *C. camphora* and putative hybrids form three clusters. The bootstrap numbers on the nodes splitting putative hybrids, *C. kanehirae* and *C. camphora* were 1 and 0.994, which are high enough to support the clustering. This implies that the putative hybrids are genetically distinct from *C. kanehirae* and *C. camphora*. The PCoA analysis of 808 SNPs represented 83.95% (sum of 65.84% + 18.12%) of the total variance (Fig. 3). The ovals around the sample points are the results of the morphological and phylogenetic analysis. *C. kanehirae*, the putative hybrids and *C. camphora* were clearly separated into three different groups. This PCoA result is consistent with phylogenetic analysis and illustrates the genetic distance of the hybrids. The *C. camphora* accessions were also divided into two subgroups, i.e., the NW and ESW populations.

**Figure 4:**
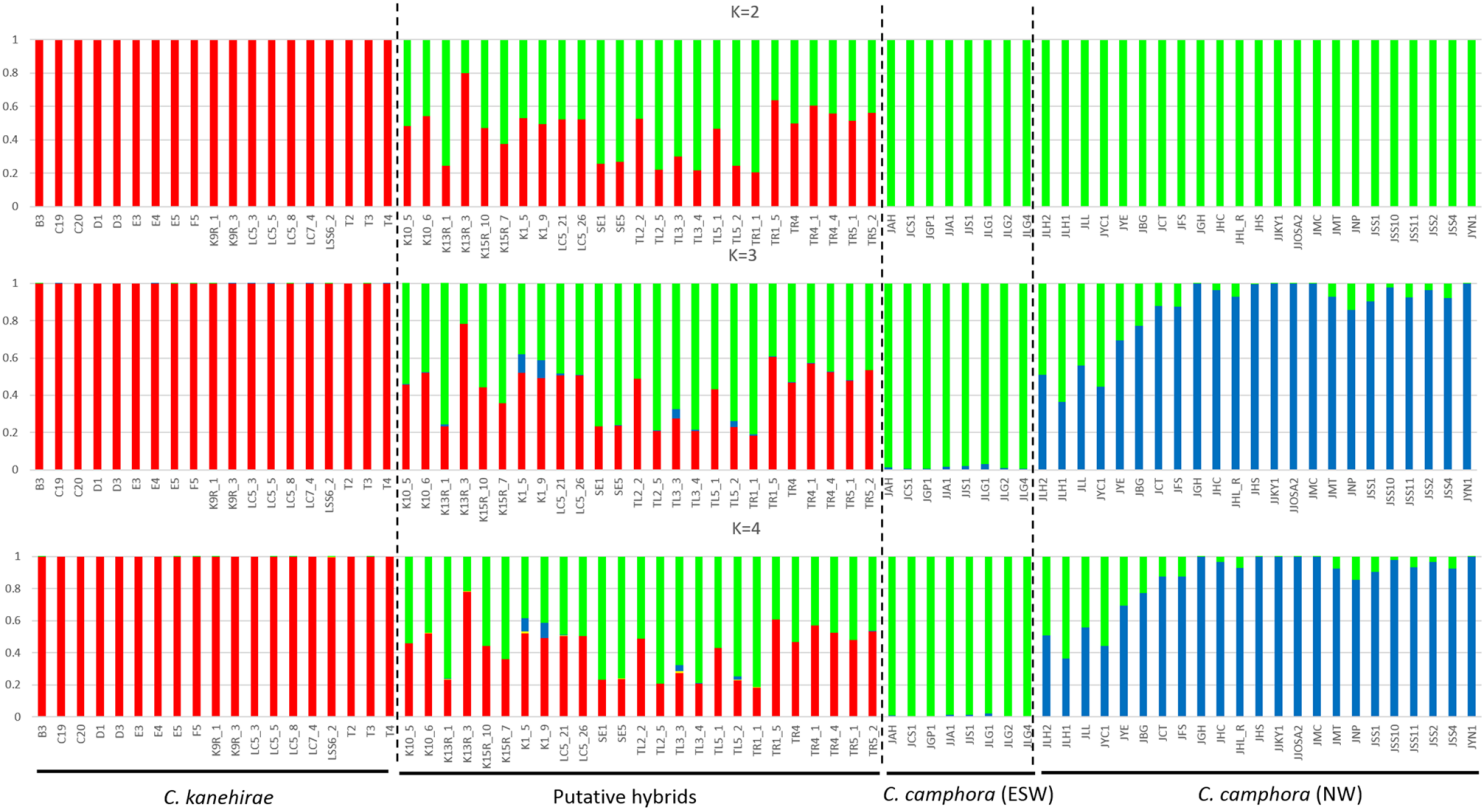
Identification and classification of hybrids. STRUCTURE results showing the proportion of the genome of every individual originating from each of the inferred clusters (K = 2); red is cluster 1 (*C. kanehirae*) and green is cluster 2 (*C. camphora*); the hybrids are mixed with red and green. STRUCTURE of K = 3, 23 accessions of the green cluster were divided into the blue cluster. All ESW *C. camphora* have high Q-values in the green cluster. STRUCTURE of K = 4, only the red cluster was divided into the yellow cluster in *C. kanehirae* and putative hybrids. The dotted lines are classified by morphological characteristics and phylogenetic analysis.

**Figure 5:**
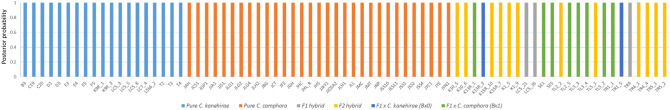
NewHybrids results with the putative pedigrees for all 75 individuals and posterior probabilities for a given class in different color bars.

All samples were included in clustering analysis with the STRUCTURE software, and the number of clusters (K) was estimated to be 2 when delta K statistics were given by the STRUCTURE Harvester^24^. We examined the genetic structure assuming different Ks between 2 and 4 (Fig. 4). The results showed that when K = 2, *C. kanehirae* and *C. camphora* are clearly separated in two groups (red and green), and all of the putative hybrids were genetic composition admixtures from both species. Fifty pure individuals (19 *C. kanehirae* and 31 of *C. camphora*) had Q-values ≥ 0.9, and 25 putative hybrids had Q-values from 0.1 to 0.9. Therefore, we classified them as hybrids. When the cluster number (K) was equal to 3, the cluster including all *C. camphora* was divided into two clusters (green and blue). When K = 4, the genetic structure only changed in the red cluster (*C. kanehirae* and putative hybrids). Hybrid individuals with Q-values close to 0.5 were interpreted to be the F1 (*C. kanehirae* × *C. camphora*) and F2 (F1 × F1). Hybrid individuals with Q-values close to 0.25 or 0.75 may be interpreted as backcrossed to *C. kanehirae* or *C. camphora* (F1 × *C. kanehirae* or F1 × *C. camphora*). In the analysis using NewHybrid software, all of the posterior probabilities of *C. camphora* and *C. kanehirae* were 1 (Fig. 5). This indicated that all of the *C. kanehirae* and *C. camphora* are pure.

## Discussion

Within this study, the InDel markers were used to rapidly and successfully detect the hybrids and their parents. InDel markers are codominant and relatively abundant, and they offer easy direct detection by PCR and subsequent gel electrophoresis^25,26^. In most angiosperms, mitochondrial DNA is maternally inherited^27^. The InDel markers in this study are suitable for detecting F1 hybrids but not F2 or backcross hybrids. The InDel data from the mitochondria also revealed that in these hybrids, *C. kanehirae* is the mother tree, and *C. camphora* is the donor parent. Taiwan was a possible site for the origin and differentiation of the *Cinnamomum* sect. *camphora*^28^. Our data also reveals that the genotypes of east and west *C. camphora* are different. This is consistent with the previous report that the *C. camphora* in eastern Taiwan is unique and genetically distinct from the *C. camphora* in western Taiwan and Japan^29^. These genotyping data are partially in accordance with previous reports. Kameyama et al. used SSR markers to calculate the genetic diversity of *C. camphora* from China, Japan and Taiwan, and the data showed that the *C. camphora* in Japan showed a high genetic diversity compared to genotypes from parts of China and Taiwan. However, the report^30^ lacked *C. camphora* individuals collected from ESW Taiwan. The topic of geographic genetic variance in *C. camphora* seems important for further investigation in the future.

In present study, we generated 529,006 SNP sequences in the SNP calling. However, the proportion of missing sites reached 0.95 in samples of JMC and JLH2, and we removed the SNPs with missing data for high accuracy. The missing data may be relative to mutations in the recognition site of the enzyme, insertion/deletion sequences or sequencing quality. Removing all of the missing data helps to ensure the accurate SNPs for further analysis, but it dramatically reduced the size of the observed GBS dataset^31^. Regarding genetic analysis, expected heterozygosity (*He*) is also known as gene diversity, which is a measurement for understanding the genetic variation in a population. In this study, the *He* values of *C. kanehirae* and *C. camphora* are much lower than those obtained in a previous report^12,30,32^, although all of the previous reports now reveal that the *C. kanehirae* had a low level of genetic diversity in Taiwan due to the overlogging and destruction or fragmentation of the natural habitat. One of the reasons explaining the lower diversity of *C. camphora* may be that only the widely distributed giant trees (estimated older than 100 years old) were collected and analyzed in this study. The expected heterozygosity (*He*) in putative hybrids was higher than those in *C. kanehirae* and *C. camphora*. The possible reason for this result is that the genomic recombination occurs in putative hybrids and then increases the genetic diversity. The hybridization may increase the genetic diversity^33,34^. The *F*_*is*_ (inbreeding coefficient) is used to gauge the strength of inbreeding. Positive *F*_*is*_ values also suggest a significant deficiency of heterozygosity in *C. kanehirae* and NW *C. camphora*, where inbreeding leads to a decrease of genetic diversity^12,32^. The *F*_*is*_ of putative hybrids was negative, implying a heterozygote excess, which is possibly due to hybridization^35^. *F*_*st*_ is the level of genetic differentiation between populations and provides the basis for a measure of genetic distance. The values of *F*_*st*_ range from 0 to 1. A zero value indicates no population structuring or subdivision. *F*_*st*_ values of 0.05 < *F*_*st*_ < 0.15 indicate moderate differentiation, values of 0.15 < *F*_*st*_ < 0.25, indicate great differentiation, and values over 0.25 indicate very great differentiation^36^. A relatively high level of genetic differentiation was observed between the *C. kanehirae* and ESW *C. camphora* (*F*_*st*_ of 0.642), this implies that there is very great differentiation between *C. kanehirae* and *C. camphora,* consistent with their being taxonomically different species. A high level of genetic differentiation between putative hybrids and *C. kanehirae* (0.304) and NW *C. camphora* (0.267) was found. Hybrids had almost moderate genetic differentiation between *C. camphora* (0.208–0.267) and *C. kanehirae* (0.304). This implies that the hybrids are equally close to *C. kanehirae* and *C. camphora* (Table 2). Interestingly, the level of genetic differentiation between putative hybrids and ESW *C. camphora* (0.208) is lower than that between putative hybrids and NW *C. camphora* (0.267). This implies that the putative hybrids are genetically closer to ESW *C. camphora* than to NW *C. camhora*. The two *C. camphora* groups (NW and ESW *C. camphora*) also show a moderate level of differentiation from each other but high genetic diversity within *C. camphora* populations^36^. This may be due to the geographical barrier of the Central Mountain Ridge in Taiwan, and thus, the natural gene flow of *C. camphora* between east and west may be difficult. Most local plant species exhibit some genetic differentiation because of the Central Mountain Ridge in Taiwan^37^. This result is consistent with the report of Professor Naonori Hirota, in which the eastern camphor trees were found to be different from the those in western Taiwan^29^. In the STRUCTURE analysis (Fig. 4), when K = 3, the green and blue indicate the samples of ESW *C. camphora* and NW *C. camphora*, respectively. This finding of two subpopulations of *C. camphora* is also in accordance with our InDel and PCoA results. The Central Mountain Ridge in Taiwan may cause reproductive isolation between eastern and western areas in Taiwan. It clearly indicated that the hybrids originated from ESW *C. camphora* in the region of eastern or southeastern Taiwan because the hybrids have a genetic composition reflecting ESW *C. kanehirae* and *C. camphora* admixture.

Figure 5 shows that three individuals (TR4, LC5_21 and LC5_26) were classified as F1. This result is consistent with the codominant InDel with double bands in Fig. 1C,F. TR4, the street tree, has the oblate fruit and back-leaf covered with a light powder-like white wax. There are other five putative hybrids (TR1, TR5, TL2, TL3 and TL5) and several *C. kanehirae* and *C. camphora* trees planted nearby TR4 (see Supplementary Fig. S3A online). *C. kanehirae* trees (TR2, TR3, TL1, and TL4) nearby TR4 did not fruit during our investigation period from 2015 to 2019. However, *C. kanehirae* trees may be in sparse bloom on the top layer of the trees, which we did not observe. *C. camphora* is mostly pollinated by insects from the order Diptera, which can carry pollen for long distances, at least 2 km, and transport viable pollen over at least 400 m^38–41^. The closest *C. camphora* trees are approximately 80 m away from TR4. Therefore, TR(L)i_j (i.e., TR4_1, TR5_2, and TL2_5) have been classified as F1 and backcross (F1 × *C. camphora* or F1 × *C. kanehirae*). LC5_21 and LC5_26 were collected from a *C. kanehirae* tree (LC5) in the nursery, and artificial embryo culture was used to produce the seedlings. Evidence supported the natural hybridization in the individuals collected in the present experiment and was consistent with the surroundings. For instance, in the surroundings of LC5, several *C. camphora* trees were planted nearby, at approximately 100 m away, and the blooming times of both trees in the collected year were simultaneous. We used artificial embryo culture to produce the siblings LC5_21 and LC5_26, and other siblings (LC5_3, 5 and 8) exhibit similar morphological characteristics to *C. kanehirae*. The hybrid individuals collected and embryo cultured from the National Museum of Natural Science (NMNS) are F2 and B × 1 or B × 0 because the collected trees there were planted very close to each other in an array along with *C. kanehirae* (see Supplementary Fig. S3B online). The *C. kanehirae* tree (K9R) at NMNS bloomed annually from 2014 to 2018. Thus, it is reasonable that the offspring of hybrids in NMNS are F2 and B × 1 or B × 0 (K13R_3). These data indicated that the hybridization events were spontaneous and random.

The molecular and morphological data of hybrids in this study supported the occurrence of hybridization, which shows that the species boundaries are partly permeable. Both the STRUCTURE and the NewHybrid analyses showed that these individuals (F1 hybrids, F2 and backcrosses to either *C. kanehirae* or *C. camphora*) were morphologically intermediate between *C. kanehirae* and *C. camphora*. Our data implied that introgression is bidirectional in these two species, especially in human-modified areas (street trees or nursery). We know that the *C. camphora* and *C. kanehirae* rarely display synchronous blossoming in natural fields at lower elevation. Thus, a premating barrier between *C. kanehirae* and *C. camphora* is reasonable. We observed the *C. kanehirae*, *C. camphora* and hybrids bloomed synchronously in the nursery and among domesticated (street) trees. It could be possible that with the unusual climate for *C. kanehirae*, the premating barrier became weak, allowing some proportion of these related species to hybridize. Our data are of relevance because the *C. kanehirae* wood has high market value for use in culturing *A. cinnamomea*. Although hybrids might be useful in breeding programs and may outcompete the parent species^17,42^, the experimental evaluation of hybrid wood quality is still necessary in the future to ensure the alternative utilization of the hybrids. Therefore, from the conservation point of view, before the evaluation of the effects in the hybrid population, it is better to limit the dispersal of the hybrids in the nursery.

## Conclusion

In this study, we provided useful InDels to quickly identify *C. kanehirae*, *C. camphora* and their F1 hybrids (*C. kanehirae* × *C. camphora*). The genetic analysis with 808 SNPs also supports the occurrence of hybridization between *C. kanehirae* and *C. camphora*. For the conservation and breeding of *C. kanehirae* and *C. camphora*, further studies are necessary to ensure the properties of hybrids, including the wood quality, adaptive ability and so on. We infer that many hybrid seedlings appear in the market and will extend to natural fields in the future. Hybrid woods may negatively affect the commercial value of pure *C. kanehirae* wood. Thus, we recommend that buffer zones be built around the seed orchard and nursery of *C. kanehirae* to avoid cross-species pollination and to preserve the purity of the species. A suitable method for the rapid molecular detection of hybrids is also necessary to preserve and control seedling production in the future.

## Material and methods

### Plant materials and DNA extraction

The details of the plant samples used in this study are listed in Supplementary Table S4 and Supplementary Fig. S4 online. All of the *C. kanehirae* leaves were originally collected from natural habitats all over of Taiwan. Twenty-nine *C. camphora* samples were collected from giant trees (predicted ages are all over 100 years old in order to avoid sampling human-planted *C. camphora*) in Taiwan, and two *C. camphora* samples were collected in Japan, while one was collected in Vietnam. Putative hybrids were collected from among street trees near the campus and museum or in the seedling nursery. The samples labeled with underscores were collected as seeds, and then, the plant was obtained by using artificial embryo culture. The method of artificial embryo culture to generate the plant from fruit trees of *C. kanehirae* and putative hybrids followed our previous publications^43,44^. Samples were labeled as follows: TR1_5 was a seed collected from a TR1 mother tree; K9R_1 and K9R_3 were the seeds collected from a K9R mother tree; TR4_1 and TR4_4 were seeds collected from TR4, and so forth. The DNA extraction of leaf samples followed the CTAB method^45^. All of the samples were collected and immediately stored in a – 80 ℃ freezer.

### Bioinformatic and genotyping analysis

DNA from the 75 plant samples was prepared for GBS and sequencing library construction following the previous report^5^. The sequencing library was sequenced with the Illumina Hi-seq 4000 paired-end sequence platform (Illumina, San Diego, CA, USA) at Genomics (Genomics Ltd., New Taipei City, Taiwan). For low-coverage sequencing, the genomic DNAs of *C. camphora* and *C. kanehirae* were sequenced with the Illumina Mi-seq paired-end sequence platform (Illumina, San Diego, CA, USA) with a MiSeq Reagent Kit V3 (600 cycles) at the National Yang-Min University, Taiwan. The paired-end libraries were finished with Illumina TruSeq DNA PCR-free sample preparation (550 bp insert size). The adapter sequences and low-quality score of the raw reads were trimmed by the CLC quality trim tool with default parameters, and all of the trimmed reads were used for de novo assembly with the CLC genome assembler (Genomics Workbench 8.0.1, CLC Inc. Aarhus, Demark). Before InDel calling, the assembly contigs of over 1500 bp were selected for further analysis. The GATK procedure was used for InDel calling. In this procedure, we used the trimmed reads of *C. kanehirae* to align the assembly contigs of *C. camphora* (JLH2 and JZS), and the trimmed reads of *C. camphora*_JLH2 were aligned to contigs of *C. camphora*_JZS; thus, three InDel calling data sets were eventually obtained. As a result of GATK, we selected the InDel length of over 20 bp with parameters of GT (1/1) and AD (0 > 5). The primers were designed by using Primer 3 software^46^, and the sequences are listed in Supplementary Table S2 online. PCR amplification was performed on a Veriti 96-Well Fast Thermal Cycler (Veriti, Applied Biosystems, Foster City, CA, USA) in a 30 µl PCR volume containing 50 ng of genomic DNA by using a Taq polymerase kit (GenetBio, Daejeon, Korea) with a 60 ℃ annealing temperature (see Supplementary Table S2 online). PCR products were run in 3.0% agarose gel electrophoresis (SFR, AMRESCO, OH, USA) and visualized by EtBr under the UV light.

### Genetic analysis and population structure

The genomic reference-based method used the GATK procedure for SNP calling with only read 1 of the sequencer outputs. The accession number of the reference genome we used is GCA_003546025.1 (from scaff001 to scaff0012 with a total of 672,841,857 bp, 92% of the reference genome) in GenBank^19^. TASSEL v5.0 was used for genotyping summary and SNP filtering^47^. For obtaining real and accurate SNP sites for further analysis, we removed the SNPs that contained missing data by using TASSEL. The GenAlEx v6.5 was used to calculate observed and expected heterozygosity for each of the nuclear SNP markers. A principal coordinate analysis (PCoA), inbreeding coefficient (*F*_*is*_) and level of genetic differentiation (*F*_*st*_) were also calculated by using GenAlEx v6.5^48^. The phylogenetic tree was constructed using MEGA 6.0 by the neighbor-joining (NJ) method with 1,000 bootstrap replicates^49^. The genetic structure was investigated using Bayesian cluster analyses with the STRUCTURE software v2.3.4^50,51^. An admixture model was employed in which correlated allele frequencies were assumed and the K-value (i.e., the number of clusters) was set from one to seven. The length of the burn-in period was set to 10,000, and the Monte Carlo Markov Chain (MCMC) model after burn-in was run for an additional 100,000 iterations. For each K, 20 replicates were run, and the best delta K was obtained by using STRUCTURE harvester online software^24^.

### Identification and classification of hybrids

For morphological trait analysis, we examined the color of the back-leaf and the fruit size in some of the *C. kanehirae*, *C. camphora* and the putative hybrids. All of the pure species were validated by codominant species-specific InDel markers (JLH2_CK InDel_4). F1, F2 (F1 × F1) and backcross (B × 0: F1 × *C. kanehirae*; B × 1: F1 × *C. camphora*) hybrids were identified using the SNP genotypic data with the software STRUCTURE and NewHybrids^50,52^. The Q-value from STRUCTURE, which shows the proportion of an individual’s genome that originates from each of the K clusters, was used as a hybrid index. Thus, for the best delta K, individuals with Q-values between 0.1 and 0.9 were considered to be hybrids or backcrosses, while individuals with Q-values ≥ 0.9 were considered to be pure species. This coincides with previous studies^2,17,53,54^. NewHybrids was used to classify individuals as pure parent species, F1s, F2s or backcrosses. NewHybrids computes the posterior probabilities that an individual belongs to these different classes. For NewHybrids, we used the default genotype categories for first and second generations of crossing and ran 100,000 sweeps of five chains started from over dispersed starting values after a burn-in period of 50,000 sweeps. Jeffrey-type priors were used for the mixing proportions and allele frequencies. To ensure the successful convergence of NewHybrids, the *C. kanehirae* (B3) and *C. camphora* (JAH) were set to individual-specific prior (z option) for pure parents.

## Supplementary information

Supplementary Information.

## Data Availability

The genotyping data of 840 SNPs obtained by GBS in this study will be available from the TFRI data Catalog, DataOne (https://metacat.tfri.gov.tw/tfri/). Thirty-two SNPs deviated from Hardy–Weinberg equilibrium were labeled with * in the table.

## References

[1] Mallet J (2005). Hybridization as an invasion of the genome. Trends Ecol. Evol..

[2] Fogelqvist J (2015). Genetic and morphological evidence for introgression between three species of willows. BMC Evol. Biol..

[3] Tu N, Junji K, Kazuyuki M (2009). Possibility of improvement in fundamental properties of wood of acacia hybrids by artificial hybridization. J. Wood Sci..

[4] Baack EJ, Rieseberg LH (2007). A genomic view of introgression and hybrid speciation. Curr. Opin. Genet. Dev..

[5] Elshire RJ (2011). A robust, simple genotyping-by-sequencing (gbs) approach for high diversity species. Curr. Opin. Genet. Dev..

[6] Wu CC, Chu FH, Ho CK, Sung CH, Chang SH (2017). Comparative analysis of the complete chloroplast genomic sequence and chemical components of *Cinnamomum micranthum* and *Cinnamomum kanehirae*. Holzforschung.

[7] Das S (2015). Genome-wide insertion-deletion (InDel) marker discovery and genotyping for genomics-assisted breeding applications in chickpea. DNA Res..

[8] Mammadov J, Aggarwal R, Buyyarapu R, Kumpatla S (2012). SNP markers and their impact on plant breeding. Int. J. Plant Genom..

[9] Renner SS (2011). Laurales. eLS.

[10] Cheng S-S (2015). Chemical polymorphism and composition of leaf essential oils of *Cinnamomum kanehirae* using gas chromatography/mass spectrometry, cluster analysis, and principal component analysis. J. Wood Chem. Technol..

[11] Huang TC (2003). Flora of Taiwan II.

[12] Liao PC (2010). Historical spatial range expansion and a very recent bottleneck of *Cinnamomum kanehirae* Hay. (Lauraceae) in Taiwan inferred from nuclear genes. BMC Evol. Biol..

[13] Lin TY (2011). Metabolite profiles for Antrodia cinnamomea fruiting bodies harvested at different culture ages and from different wood substrates. J. Agric. Food Chem..

[14] Tzeng YM, Geethangili M (2011). Review of pharmacological effects of Antrodia camphorata and its bioactive compounds. Evid. Complement. Altern. Med..

[15] Chang SH, Ho CK, Lin ST (2014). Production of *Cinnamomum kanehirae* clone and seed seedling. Taiwan For. Res. Newsl..

[16] Chung JD, Chen SY, Chien CT (2012). *Cinnamomum kanehirae* seed orchard. Taiwan For. Res. Newsl..

[17] Levänen R, Thulin CG, Spong G, Pohjoismäki JLO (2018). Widespread introgression of mountain hare genes into Fennoscandian brown hare populations. PLoS ONE.

[18] Wu CC, Ho CK, Chang SH (2016). The complete chloroplast genome of *Cinnamomum kanehirae* Hayata (Lauraceae). Mitochondrial. DNA A.

[19] Chaw SM (2019). Stout camphor tree genome fills gaps in understanding of flowering plant genome evolution. Nat. Plants.

[20] Chen B, Cole JW, Grond-Ginsbach C (2017). Departure from Hardy Weinberg Equilibrium and genotyping error. Front. Genet..

[21] Miyagawa T (2008). Appropriate data cleaning methods for genome-wide association study. J. Hum. Genet..

[22] Lin TP (1993). *Cinnamomum kanehirae* Hay. and *Cinnamomum micranthum* (Hay.) Hay. Bull. Taiwan Res. Inst..

[23] Liu HY, Yang YP, Lu SY, Shih BL (2000). Manual of Taiwan Vascular Plants.

[24] Earl DA, von Holdt BM (2012). STRUCTURE HARVESTER: a website and program for visualizing STRUCTURE output and implementing the Evanno method. Conserv. Genet. Resour..

[25] Niihama M, Mochizuki M, Kurata N, Nonomura K (2015). PCR-based INDEL markers co-dominant between *Oryza sativa*, japonica cultivars and closely-related wild Oryza species. Breed. Sci..

[26] Pacurar DI (2012). A collection of INDEL markers for map-based cloning in seven *Arabidopsis* accessions. J. Exp. Bot..

[27] Reboud X, Zeyl C (1994). Organelle inheritance in plants. Heredity.

[28] Fujita Y (1952). *Cinnamomum camphora* Sieb. and its allied species. Their inter-relations considered from the view-points of species characters, chemical constituents, geographical distributions and evolution. Bot. Mag. Tokoyo.

[29] Youngman BJ (1952). Professor Naonori Hirota ’ s work on camphor trees. Kew Bull..

[30] Kameyama Y, Furumichi J, Li J, Tseng YH (2017). Natural genetic differentiation and human-mediated gene flow: the spatiotemporal tendency observed in a long-lived *Cinnamomum camphora* (Lauraceae) tree. Tree Genet. Genomes.

[31] Sciences, N. A. of. *In the Light of Evolution: Volume X: Comparative Phylogeography*. (The National Academies Press, Taipei, 2017).

[32] Hung KH, Lin CH, Ju LP (2017). Tracking the geographical origin of timber by DNA fingerprinting: a study of the endangered species *Cinnamomum kanehirae* in Taiwan. Holzforschung.

[33] Ball JW, Robinson TP, Bovill J, Byrne M, Nevill PG (2020). Fine-scale species distribution modelling and genotyping by sequencing to examine hybridisation between two narrow endemic plant species. Sci. Rep..

[34] Zalapa JE, Brunet J, Guries RP (2010). The extent of hybridization and its impact on the genetic diversity and population structure of an invasive tree, *Ulmus pumila* (Ulmaceae). Evol. Appl..

[35] Zhang J (2017). Effect of domestication on the genetic diversity and structure of *Saccharina japonica* populations in China. Sci. Rep..

[36] Balloux & Lugon-Moulin (2002). The estimation of population differentiation with microsatellite markers. Mol. Ecol..

[37] Cheng YP, Hwang SY, Lin TP (2005). Potential refugia in Taiwan revealed by the phylogeographical study of *Castanopsis carlesii* Hayata (Fagaceae). Mol. Ecol..

[38] Fan YB (2006). The Floral and Pollination Biology of Lauraceae in Taiwan.

[39] Corlett RT (2001). Pollination in a degraded tropical landscape: a Hong Kong case study. J. Trop. Ecol..

[40] Rader R, Edwards W, Westcott DA, Cunningham SA, Howlett BG (2011). Pollen transport differs among bees and flies in a human-modified landscape. Divers. Distrib..

[41] Inouye DW, Larson BMH, Ssymank A, Kevan PG (2015). Files and flowers III: ecology of foraging and pollination. J. Pollinat. Ecol..

[42] Rollo A (2016). Genetic diversity and hybridization in the two species Inga ingoides and Inga edulis: potential applications for agroforestry in the Peruvian Amazon. Ann. For. Sci..

[43] Chang SH (2015). Somatic embryogenesis and plant regeneration from immature embryo cultures of *Cinnamomum kanehirae*. Taiwan J. For. Sci..

[44] Chang SH, Ho CK, Tsay JY (2002). In Vitro Culture of *Cinnamomum kanehirae* Hayata. Taiwan J. For. Sci..

[45] Doyle J, Doyle J (1990). Isolation of plant DNA from fresh tissue. Focus.

[46] Untergasser A (2012). Primer3: new capabilities and interfaces. Nucleic Acids Res..

[47] Bradbury PJ (2007). TASSEL: software for association mapping of complex traits in diverse samples. Bioinformatics.

[48] Peakall R, Smouse PE (2012). GenALEx 6.5: Genetic analysis in Excel. Population genetic software for teaching and research-an update. Bioinformatics.

[49] Tamura K, Stecher G, Peterson D, Filipski A, Kumar S (2013). MEGA6: molecular evolutionary genetics analysis version 6.0. Mol. Biol. Evol..

[50] Pritchard JK, Stephens M, Donnelly P (2000). Inference of population structure using multilocus genotype data. Genetics.

[51] Hubisz MJ, Falush D, Stephens M, Pritchard JK (2009). Inferring weak population structure with the assistance of sample group information. Mol. Ecol. Resour..

[52] Anderson EC, Thompson EA (2002). A model-based method for identifying species hybrids using multilocus genetic data. Genetics.

[53] Catherine IC (2011). Mountain pine beetle host-range expansion threatens the boreal forest. Mol. Ecol..

[54] De La Torre A, Ingvarsson PK, Aitken SN (2015). Genetic architecture and genomic patterns of gene flow between hybridizing species of *Picea*. Heredity.

