# Supplementary material for: Identification of hybridization and introgression between Cinnamomum kanehirae Hayata and C. camphora (L.) Presl using genotyping-by-sequencing
Source: Sci Rep. 2020 Sep 29;10:15995. doi: 10.1038/s41598-020-72775-0 (PMC7525239; doi:10.1038/s41598-020-72775-0)
Supplement: Supplementary file 1 — Supplementary Information. [file 41598_2020_72775_MOESM1_ESM.pdf]

Original Paper

Identification of hybridization and introgression between *Cinnamomum kanehirae* Hayata and *C. camphora* (L.) Presl using genotyping-by-sequencing

Chia-Chen Wu[1, 2], Shu-Hwa Chang[1], Chih-Wei Tung[3], Cheng-Kuen Ho[1], Yolanda Gogorcena[4] and Fang-Hua Chu[2 *]

[1] Silviculture Division, Taiwan Forestry Research Institute, Council of Agriculture, Executive Yuan, Taiwan

[2] School of Forestry and Resource Conservation, National Taiwan University, Taiwan

[3]Department of Agronomy, National Taiwan University, Taiwan

[4]Laboratory of Genomics, Genetics and Breeding of Fruit Trees and Grapevines, Experimental Station of Aula Dei-CSIC, Spain

*Author for correspondence: fhchu@ntu.edu.tw

Supplementary Tables and Figures

Supplementary Table S1. The statistical data of low-coverage sequencing and *de novo* assembly

	*C. kanehirae*_S99	*C. camphora*_JLH2	*C. camphora*_JZS
NGS raw reads	16,233,870	13,406,586	12,928,238
Raw sequencing bases (bp)	4,886,394,870	3,855,672,343	3,846,225,943
Reads after trimmed	16,233,870	12,966,564	12,886,530
Clean bases after trimmed (bp)	4,833,754,899	1,880,522,918	2,947,203,453
de novo assembly summary by CLCbio			
N50 (bp)	1,583	1,470	552
Average length (bp)	920	1,259	505
Count	492,338	170,070	409,869

Supplementary Table S2. List of Indel primer sequences and BLASTn results.

Entry	InDel_names	Forward Primer sequences	Reverse Primer sequences	Validation	BLASTn of aligned contig
**1**	JLH2_CK Indel_4	5'-CGTTTCAAGATTGTTTGGCCC-3'	5'-CGCAAGTTCATTTTCCTCATGC-3'	**polymorphic**	*Solanum tuberosum* chromosome 5 clone RH072I03, complete sequence
**2**	JLH2_ZS Indel_2	5'-CGAAGTCATCACCCATTGTCC-3'	5'-CGCTAGGACTCAGGTAAGCC-3'	**polymorphic**	no hit match
**3**	JLH2_ZS Indel_3	5'-CCTCAATAAAAGGGCTGGCC-3'	5'-ACCGATTTGAGATGCCATCA-3'	**polymorphic**	no hit match
**4**	ZS_CK indel_1	5'-CATTTCTCGCCTCAACCCAG-3'	5'-AACCGGCCACTAGTTACGAA-3'	**polymorphic**	*Liriodendron tulipifera* mitochondrion, complete genome
**5**	ZS_CK indel_2	5'-ATCCTTCAACTCCCCTAGGC-3'	5'-TCCTCGTGTCACCAGTTCAA-3'	**polymorphic**	*Persea americana* voucher SY01359 chloroplast, complete genome
**6**	ZS_CK indel_3	5'-TCAATTAGGTGCGGGAAGGT-3'	5'-GGGTAAGCAGGACTACGAGT-3'	**polymorphic**	*Castilleja paramensis* mitochondrion, complete genome
**7**	ZS_CK indel_5	5'-TGGCTCCAAAGTGTCATATTGAG-3'	5'-GTGCTACAGTGATTTCGACCA-3'	**polymorphic**	no hit match
**8**	JLH2_CK Indel_1	5'-TGTCCAACCCATGCTTTGAG-3'	5'-CTTCAATCCTTGGCAGACACA-3'	monomorphic	*Persea americana* PaGAL4 mRNA for beta-D-galactosidase, complete cds
**9**	JLH2_CK Indel_2	5'-AACGCAAACAAACAGTCCCT-3'	5'-CCTTATTTTGGATGGGTGAGGC-3'	monomorphic	PREDICTED: *Nelumbo nucifera* alpha-mannosidase (LOC104590020), transcript variant X3, mRNA
**10**	JLH2_CK Indel_3	5'-TTCTTGGGTTTGGCATGTGG-3'	5'-TTACGCCCAAGAGCAAAGAG-3'	monomorphic	no hit match
**11**	JLH2_CK Indel_5	5'-CACCAGCACTTGAAGGAACC-3'	5'-CCTAGAAGCACGTGATTGGC-3'	monomorphic	no hit match
**12**	JLH2_ZS Indel_1	5'-TGTGATGGAACGAACTCTTTGT-3'	5'-AGCTGCCAATAGAGACAAAACA-3'	monomorphic	no hit match

**13**	JLH2_ZS Indel_4	5'-CAACGTGTTTGTGAGAGTGGT-3'	5'-CAAGGGGTGCAAAGAAGATTG-3'	monomorphic	PREDICTED: *Dendrobium catenatum* 14-3-3-like protein D (LOC110116516), mRNA
**14**	JLH2_ZS Indel_5	5'-TGACTCGAGTTGTTGGTAGGT-3'	5'-GCCCATTTCGACCATCCATT-3'	monomorphic	no hit match
**15**	ZS_CK indel_4	5'-TGTGGGGAAGAGGAGATGAA-3'	5'-TCTCCACCATCCATGCTCAA-3'	monomorphic	no hit match

Supplementary Table S3. Morphological measurements of fruits in *C. kanehirae,* putative hybrids and *C. camphora*.

	C. kanehirae	putative hybrid	*C. camphora*
Length of Fruit (cm)	0.91±0.13	1.1±0.17	0.83±0.13
Width of Fruit (cm)	1.19±0.14	1.28±0.12	0.84±0.15
Ratio of L/W	0.762	0.867	0.992

Supplementary Table S4. List of plant materials, locations, and experimental procedure and use in this study. The numbers are the same as the numbers in parentheses in Supplementary Fig. S1.

Experimental use	Entry	Label name/species	Description	Collection locations
Low-coverage sequencing, GBS and InDel	1	*JZS/C. kanehirae*	Fresh leaves	Except for 35 (Kyoto, Japan), 36 (Osaka, Japan) and 37 (Hội An, Vietnam), the others were collected in Taiwan.
	2	*S99/C. camphora*		
	3	*JLH2/C. camphora*		
GBS, InDel	4	*LSS6_2/C. kanehirae*	Fresh leaves, artificial embryo-culture from scion garden	
	5	*K9R_1, 3/C. kanehirae*		
	6	*LC5_3, 5, 8; LC7_4/C. kanehirae*		
	7	*B3/C. kanehirae*		
	8	*C19, 20/C. kanehirae*		
	9	*D1, 3/C. kanehirae*		
	10	*F5/C. kanehirae*		
	11	*T2, 3, 4/C. kanehirae*		
	12	*E3, 4, 5/C. kanehirae*		
	13	*JGH/C. camphora*	Fresh leaves	
	14	*JHS/C. camphora*		
	15	*JHC/C. camphora*		
	16	*JMC/C. camphora*		
	17	*JMT/C. camphora*		

	18	*JHL_R/C. camphora*		
	19	*JSS1, 2, 4, 10, 11/C. camphora*		
	20	*JCT/C. camphora*		
	21	*JNP/C. camphora*		
	22	*JFS/C. camphora*		
	23	*JLL/C. camphora*		
	24	*JBG/C. camphora*		
	25	*JYE/C. camphora*		
	26	*JYC1/C. camphora*		
	27	*JLH2/C. camphora*		
	28	*JLH1/C. camphora*		
	29	*JAH/C. camphora*		
	30	*JLG1, 2, 4/C. camphora*		
	31	*JCS1/C. camphora*		
	32	*JGP1/C. camphora*		
	33	*JJA1/C. camphora*		
	34	*JJS1/C. camphora*		
	35	*JJKY1/C. camphora*		
	36	*JJOSA2/C. camphora*		
	37	*JYN1/C. camphora*		

	38	*TL2_2, 5; TL3_3, 4; TL5_1, 2; TR1_1, 5; TR4, TR4_1, 4; TR5_1, 2/putative hybrids*	Fresh leaves, artificial embryo-culture from scion garden	
	39	*K10_5, 6; K13R_1, 3; K15R_7, 10; K1_5, 9/putative hybrids*		
	40	*LC5_21, 26/putative hybrids*		
	41	*SE1, 5/putative hybrids*		

Supplementary Table S5. List of the sample abbreviations in the electrophoresis gel (Fig. 1).

Figure 1	
A	Lane1_16: K1_5, K1_9, K10_5, K10_6, TR4, K1, K13R_1, K13R_3, K15R_7, K15R_10, LC5_21, LC5_26, SE1, SE2, JJA1 and JZS
B	Lane1_16: C19, C20, D1, D3, E3, E4, E5, F5, JAH, JCS1, JGP1, JJA1, JCT, JFS, JGH, JHC and TR4
C (the hybrids here all are F1)	Lane1_16: K1, K7, K8, K10, K13R, TR1, K15R, TL2, TL3, TR1, LC5_21, LC5_26, LC5, T2, JJL1 and JZS
D	Lane1_16: C19, C20, D1, D3, E3, E4, E5, F5, JAH, JCS1, JGP1, JJA1, JCT, JFS, JGH, JHC, JLH1, JLL, JMC, JMT, JNP, JSS10, JSS11 and JSS1
E (the hybrids here all are F2 or backcross)	Lane1_16: C20, D1, K1_5, K1_9, K10_5, K10_6, K13R_1, K13R_3, K15R_10, SE1, SE5, TL2_2, TL2_5, TL3_3, JJA1 and JZS.
F (except for TR4, the hybrids here all are F2 or backcross)	Lane1_16: TL2_2, TL2_5, TL3_3, TL4_4, TL5_1, TL5_2, TR1_1, TR1_5, TR4, TR4_1, TR4_4, TR5_1, TR5_2, SE5, JJA1 and JZS.
G	Lane1_8 (*C. kanehirae*)：C19, C20, D1, D3, E5, F5, T1, T5<br>Lane9_16 (ESW *C. camphora*)：JJL1, JJL2, JFL1, JCS1, JYC1, JAH, JJA1, JJA2<br>Lane17_24 (NW *C. camphora*)：JMC, JMT, JHL_R, JSS2, JSS4, JJKC, JTP2, JWF

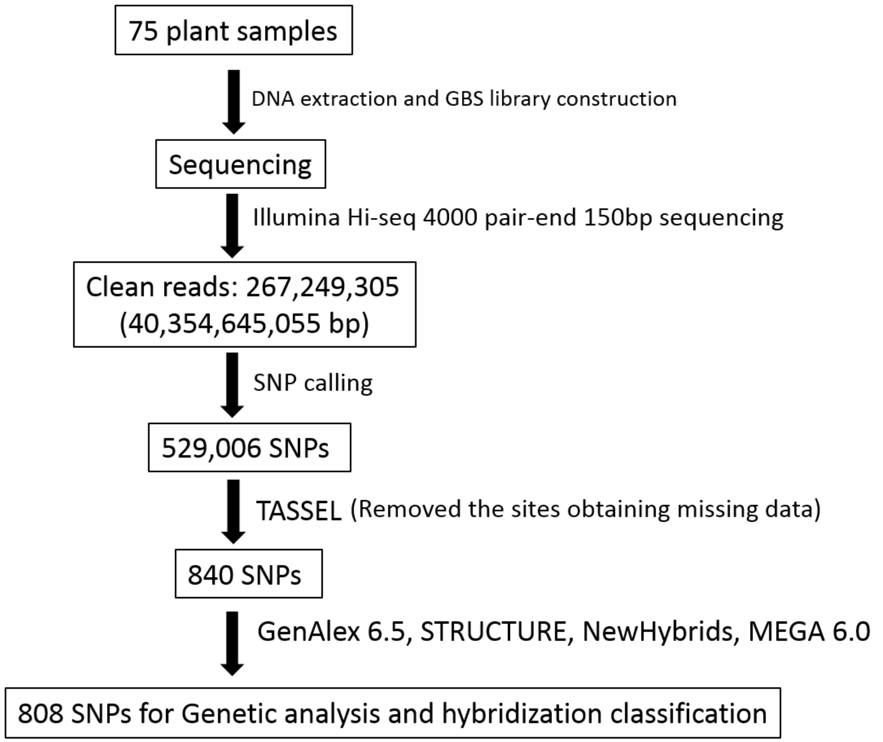

Supplementary Figure S1. Workflow of the SNP genotyping.

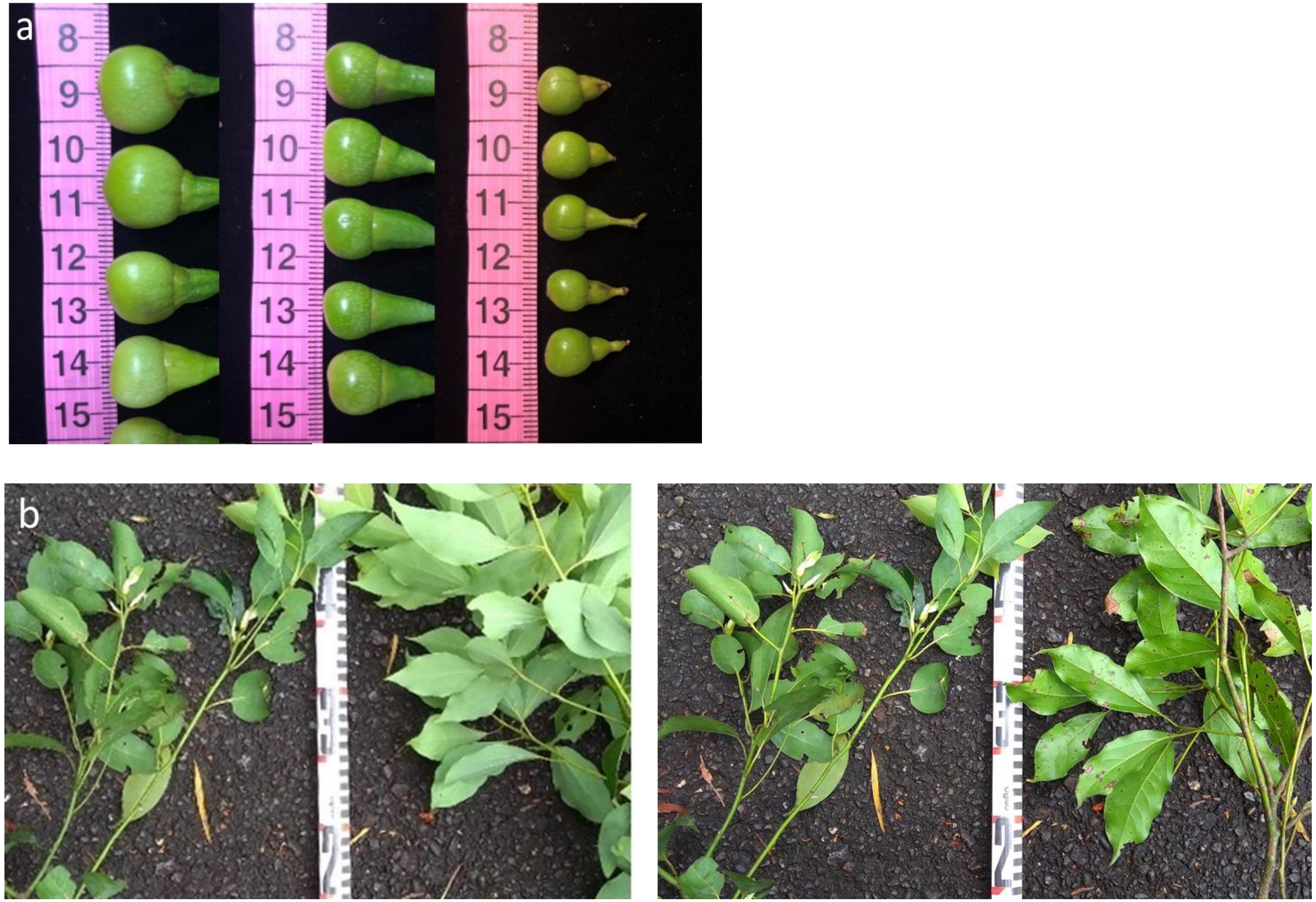

Supplementary Figure S2. (a) Photos of the fruit shape (scale: cm). (b) Photos of the back-leaves. Order (from left to right): hybrids, *C. camphora*, hybrids and *C. kanehiare* (scale: cm).

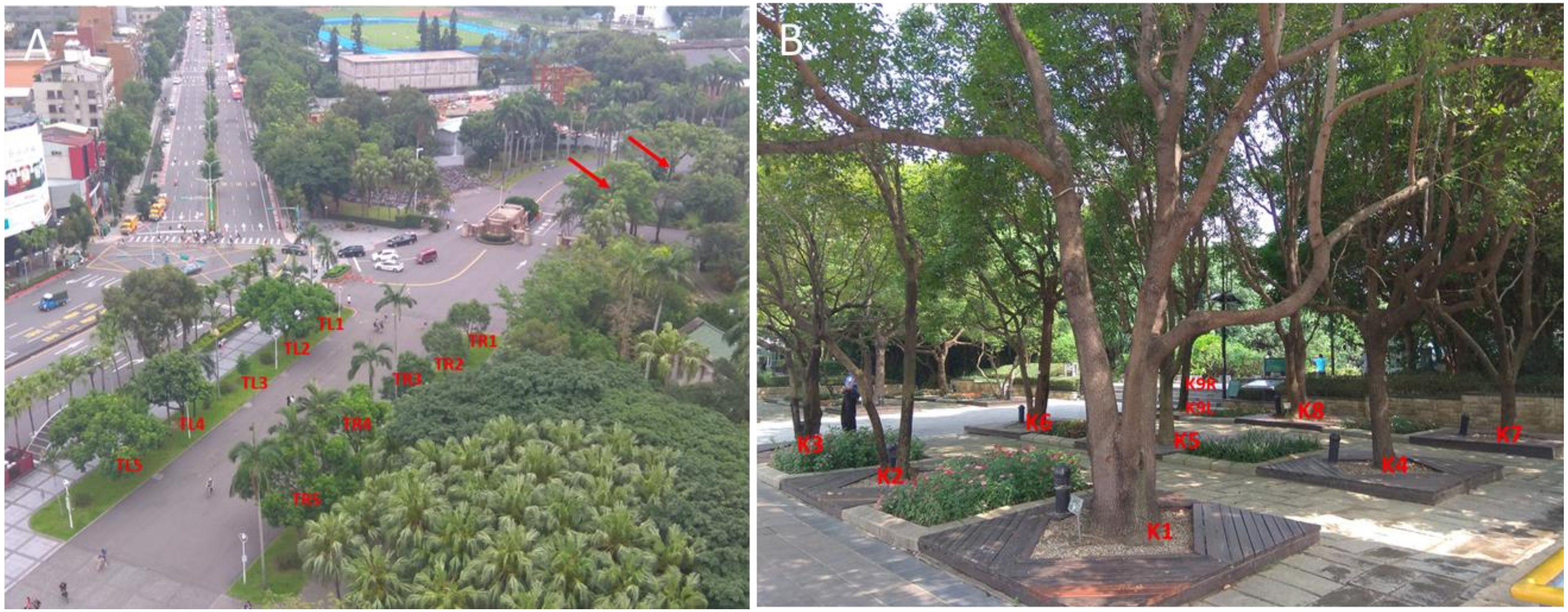

Supplementary Figure S3. Photos indicate the locations of hybrids in the street trees. (A) National Taiwan University front entrance in Taipei, Taiwan. The red arrows point the *C. camphora* trees (B) National Museum of Natural Science.; 10 trees (*C. kanehirae* and hybrids) were planted in the square.

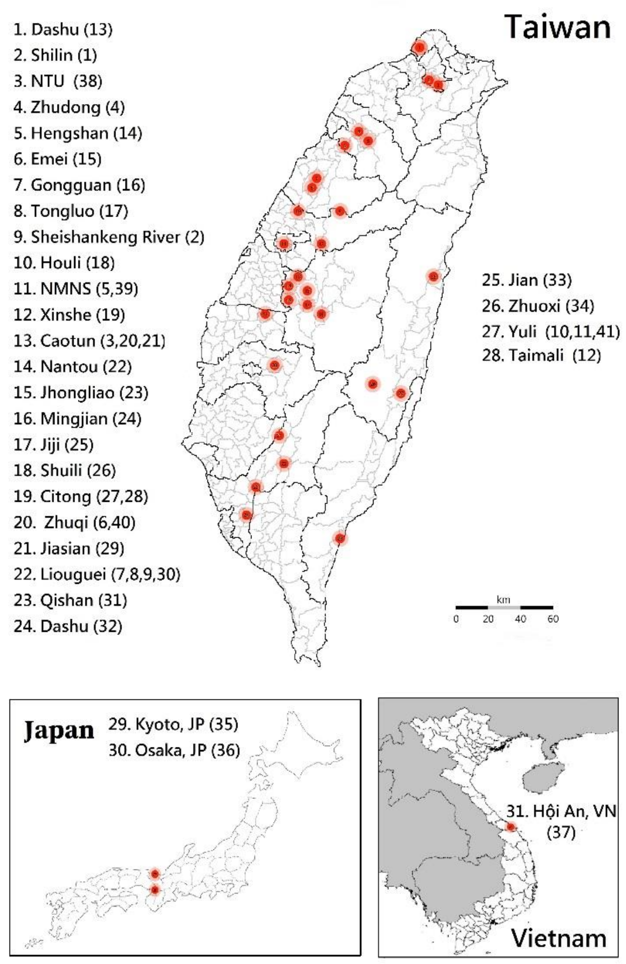

Supplementary Figure S4. Locations of the plant material sampling sites on the map (numbers in parentheses after the location correspond to the samples presented in Supplementary Table S4).
